# Performance of honey bee colonies under a long‐lasting dietary exposure to sublethal concentrations of the neonicotinoid insecticide thiacloprid†

**DOI:** 10.1002/ps.4547

**Published:** 2017-03-28

**Authors:** Reinhold Siede, Lena Faust, Marina D Meixner, Christian Maus, Bernd Grünewald, Ralph Büchler

**Affiliations:** ^1^ Landesbetrieb Landwirtschaft HessenBieneninstitut KirchhainKirchhainGermany; ^2^ Institut für Bienenkunde, Oberursel, Polytechnische Gesellschaft, Fachbereich BiowissenschaftenGoethe‐Universität Frankfurt am MainOberurselGermany; ^3^ Bayer Bee Care CentreBayer AGMonheimGermany

**Keywords:** honey bee colony, performance, thiacloprid, neonicotinoids, chronic exposure

## Abstract

**BACKGROUND:**

Substantial honey bee colony losses have occurred periodically in the last decades. The drivers for these losses are not fully understood. The influence of pests and pathogens are beyond dispute, but in addition, chronic exposure to sublethal concentrations of pesticides has been suggested to affect the performance of honey bee colonies. This study aims to elucidate the potential effects of a chronic exposure to sublethal concentrations (one realistic worst‐case concentration) of the neonicotinoid thiacloprid to honey bee colonies in a three year replicated colony feeding study.

**RESULTS:**

Thiacloprid did not significantly affect the colony strength. No differences between treatment and control were observed for the mortality of bees, the infestation with the parasitic mite Varroa destructor and the infection levels of viruses. No colony losses occurred during the overwintering seasons. Furthermore, thiacloprid did not influence the constitutive expression of the immunity‐related hymenoptaecin gene. However, upregulation of hymenoptaecin expression as a response to bacterial challenge was less pronounced in exposed bees than in control bees.

**CONCLUSION:**

Under field conditions, bee colonies are not adversely affected by a long‐lasting exposure to sublethal concentrations of thiacloprid. No indications were found that field‐realistic and higher doses exerted a biologically significant effect on colony performance. © 2017 The Authors. *Pest Management Science* published by John Wiley & Sons Ltd on behalf of Society of Chemical Industry.

## 1 INTRODUCTION

Thiacloprid is an insecticide belonging to the chemical class of neonicotinoids. These substances show structural similarities to nicotine and first came to the market in 1991, with imidacloprid as the first substance to be commercialised. Since then, further neonicotinoids, such as acetamiprid, clothianidin, thiamethoxam and thiacloprid, have taken a large share of the insecticide market. In 2014, around 20% of all domestically traded insecticides in Germany belonged to the neo nicotinoid group.^1^ Of the insecticides exported from Germany, they account for over 50% of the market.^1^ Thiacloprid is used as an active substance in products for foliar applications to control pests in orchards, arable crops, vegetable production and other specialised crops, and as a seed treatment in maize. In various countries in Northern, Western and Central Europe, an important area of application for products containing thiacloprid is the foliar treatment of oilseed rape (*Brassica napus*) at early flowering. Treatments during full blossom of the crop are permitted in some countries, as thiacloprid is characterised by a low to moderate toxicity to bees, and applications during bloom are consequently classified as safe for bees.^2^ Honey bees can relatively quickly detoxify neonicotinoids such as thiacloprid that contain a cyano substitution in their imidazolidine ring.^3^ In contrast, nitrosubstituted neonicotinioids, such as imidacloprid, are not as quickly metabolised by the detoxification system of the bees, and therefore show a substantially higher intrinsic toxicity to individual honey bees (e.g. imidacloprid: 3.7 to <104 ng bee^−1^; LD_50_ 48 h after oral uptake). The intrinsic toxicity of thiacloprid to bees is lower by a factor of ca 1000 (17.3 µg bee^−1^; LD_50_ 48 h after oral uptake).^4^ However, owing to the low to moderate toxicity of thiacloprid, the substance has frequently been applied to flowering crops, meaning that foragers collecting nectar and pollen from treated crops may carry substantial amounts of thiacloprid residues back to their hives. Indeed, thiacloprid residues have been found in stored honey and in bee bread containing oilseed rape pollen.^5^, ^6^, ^7^ Consequently, colonies might be exposed to sublethal concentrations of thiacloprid over long periods. Several studies have investigated the effects of both toxic and sublethal concentrations of thiacloprid on individual honey bees, demonstrating that thiacloprid can affect neuronal functions as well as orientation and homing behaviour,^8^ survival under starvation stress^9^ and pathological stress.^10^, ^11^ However, the relevance of long‐lasting exposure of thiacloprid to colonies has so far not been well understood. In this study, we evaluated possible effects of thiacloprid on free‐ranging colonies under field‐realistic conditions in a replicated design over three consecutive years. Exposure to the test substance concentrations was achieved by in‐hive feeding.

## 2 MATERIALS AND METHODS

### 2.1 Study design

Over three years, from July 2011 to May 2014, three groups of ten *Apis mellifera carnica* colonies each were set up for the tests. The experiment was replicated 3 times between 2011 and 2013. Exposure to thiacloprid was achieved by in‐hive feeding of spiked sugar syrup which was administered in plastic hive top feeders. Control colonies (C) were provided with syrup containing the solvent acetone without thiacloprid. Treatment groups received syrup containing either 0.2 mg thiacloprid L^−1^ (T1) or 2 mg thiacloprid L^−1^ (T2). The colonies were set up in an apiary situated in an agricultural area close to Kirchhain, Germany. During the exposure period, no major bee‐attractive crops were flowering within a 3 km flight radius of the hives. Colonies of each treatment group were placed in rows. Each block of hives was separated from the other treatment groups by a distance of approximately 20 m. The entrances of the hives were oriented towards the east. Each year, new colonies with thoroughly cleaned hive equipment were used to ensure optimised standardisation. Heat‐stable material as wooden hive boxes, bottom boards, supers and queen excluders were scraped and cleaned with hot‐water, high‐pressure cleaners. Plastic parts were cleaned with cold NaOH lye. Frames were boiled in hot NaOH lye and filled with wax foundations. All material was colour coded and only reused in the corresponding treatment groups. The placement of the blocks of the treatment groups rotated over the three years.

### 2.2 Colony management

Each year at the beginning of July, 30 shook swarms were obtained from a commercial beekeeper. The shook swarms were weighed, equilibrated to 2 kg bees, queened with 4‐week‐old sister queens and treated against *Varroa destructor* with coumaphos (Perizin^®^). A few weeks before the beginning of the experiment, all queens had been mated at the mating station of Gehlberg (Germany). The swarms were kept in a cool room for 48 h and were then moved to the apiary. The colonies were set up in hives with eight frames of wax foundation and two empty drawn combs, and were immediately fed with 5 L of sugar syrup. After 7 days a further 5 L of syrup was provided, and thereafter feeding continued with 5 L syrup aliquots at intervals of 21 days. At the end of the feeding period, each colony had been provided with a total of 25 L of syrup, which was enough food to carry them through the winter. In December, the coumaphos treatment against *Varroa* was repeated. In spring, an additional brood chamber, a honey super and one or two drone combs were added according to colony development. In cases of queen losses, sister queens of a reserve stock were added to the affected colonies.

### 2.3 Exposure to thiacloprid

Two nominal thiacloprid concentrations were administered to the colonies of the respective treatment groups, T1 with 0.2 mg thiacloprid L^−1^, and T2 with 2 mg thiacloprid L^−1^. Dosing was derived from realistic field exposure data: according to findings of the German Bee Monitoring,^6^ thiacloprid residue levels of ca 0.2 mg kg^−1^ were high‐end concentrations in bee bread samples collected from bee hives in Germany, and represented a realistic worst‐case scenario, and 2 mg kg^−1^ was a tenfold increased concentration in order to depict an unrealistic worst‐case scenario. Thiacloprid (content of active ingredient 98.3% w/w certified by Bayer AG laboratory) was obtained from Bayer AG. Each year, stock solutions of 2 mg thiacloprid mL^−1^ acetone and 20 mg thiacloprid mL^−1^ acetone were prepared in glass flasks and stored in a dark and cool room (∼15 °C) until use. Several days before feeding, 5.4 mL of each stock solution or 5.4 mL of pure acetone was added to 54 L of sugar syrup (Ambrosia^®^; 0.73 kg sugar L^−1^) to obtain syrup for controls without thiacloprid, syrup containing 0.2 mg thiacloprid L^−1^ (≈0.139 mg kg^−1^) for group T1 and syrup containing 2 mg thiacloprid L^−1^ (≈1.39 mg kg^−1^) for group T2. The mixtures were thoroughly stirred for 30 min with a whisk at high speed. From each mixture, samples were retained for chemical analysis. Samples were sent to a commercial laboratory (Eurofins Dr Specht, Hamburg, Germany) for thiacloprid content determination by the QuEChERS method according to DIN EN 15662:2009. The analytically confirmed concentrations in the feeding syrup were slightly below the targeted concentrations. In syrup for the control group, no thiacloprid was detectable (number of analysed syrup samples *N* = 11). In syrup for group T1, a mean of 0.125 mg thiacloprid kg^−1^ (≈0.178 mg L^−1^; minimum 0.04 mg kg^−1^; maximum 0.19 mg kg^−1^; *N* = 15) was found, while syrup for T2 contained 1.005 mg thiacloprid kg^−1^ (≈1.437 mg L^−1^; minimum 0.110 mg kg^−1^; maximum 1.800 mg kg^−1^; *N* = 15).

### 2.4 Pathological characterisation of the colonies

Approximately 40 g of living bees were brushed off the outer combs of each colony at the beginning of August [4 weeks after initiation (WAI) of the experiment], in mid‐September (10 WAI) and at the beginning of October (13 WAI) of each year. Samples were weighed. Bees were shaken in soapy water to remove *Varroa* mites, and detached mites were retained by filtering the suspension through a honey‐strainer double sieve. The detached *Varroa* mites were counted.^12^ In addition, 60 bees per colony were sampled from the outer combs in July, September and March for *Nosema* spp. The numbers of spores of *Nosema* spp. were microscopically determined with the help of a haemocytometer.^13^ In the middle of September of each year, a further ten bees were collected from the outer combs for virus analysis. Nucleic acids (NAs) were extracted with QIAamp Viral RNA Mini kit according to the manufacturer's instructions. Extracts were checked photometrically for purity and yield of NAs. Preparations were diluted to 20 ng NAs µL^−1^ before use in PCR. One‐step qRT‐PCRs (QuantiTect SYBR Green RT‐PCR; Qiagen, Hilden, Germany) were set up with diagnostic primers for ABPV (oligonucleotides A and B,^14^ DWV^15^ and CBPV^16^). The housekeeper Rp49 was used as a reference gene and amplification control. It was amplified with primers according to de Miranda and Fries.^17^ PCR conditions corresponded to common standards. Heavily loaded field extracts were used as positive controls. Pure water instead of RNA extracts were used as negative controls. Amplicons were checked by melting curve analysis and by separation in agarose gels, or, alternatively, by automated gel electrophoresis with a QIAxcel device (Qiagen).

### 2.5 Determination of immunological parameters

Thirty seven‐day‐old worker bees were collected from each colony at the end of August/beginning of September of each year. To obtain seven‐day‐old worker bees, wire cages were fixed on combs with emerging brood. One day later, the freshly emerged bees were collected, marked with a coloured dot and released back into their respective colonies. Seven days later, ten marked bees per colony were collected into metal cages, brought to the laboratory and supplied with either pure sugar syrup (control bees) or syrup containing 0.2 mg thiacloprid L^−1^ for bees from group T1 and syrup containing 2 mg thiacloprid L^−1^ for bees from group T2. The next day, five bees per cage were anaesthetised on ice, injected with 3 µL of a suspension containing spores and cells of *Paenibacillus larvae* (OD_600_ = 1.4) and transferred to a new cage. One day later, three of the injected and three of the non‐injected bees were shock frozen on dry ice and stored at −80 °C until extraction of RNA. From each individual bee, RNA was extracted using the RNeasy Mini kit (Qiagen) according to the manufacturer's recommendations. Yield and purity of the RNA extracts were photometrically measured, and they were diluted to 20 ng RNA µL^−1^. cDNA was prepared with 5 µL of each dilution, hexamers (1 µm) and Omniscript reverse transcriptase (Qiagen) according to the manufacturer's protocol. Then, 1 µL of cDNA of each transcript was added to 24 µL of PCR mastermix (Quantitect SYBR Green PCR Mix; Qiagen) containing either the primers Rp49‐qF and Rp49‐qB^17^ or hymenopt F and B.^18^ Real‐time PCR was performed on a BioRad myiQ^®^ device. Products of amplification were checked by analysis of melting curves and separation of the products either in agarose gels or on an automated gel device (QiAxcel; Qiagen).

### 2.6 Residue analysis

Samples for chemical analysis of thiacloprid and its metabolite thiacloprid‐amide were obtained from concentrated syrup stored in combs and from bee bread. Sampling was done mid‐September until mid‐October and at the end of March until mid‐May. Thiacloprid and its amide metabolite content in stored syrup and bee bread were determined by Bayer AG,^19^ with modifications of the cited method. In brief, 0.5 g of the sample material was extracted in 10 mL of methanol/water (3/1 v/v) and 0.1 mL formic acid L^−1^. After centrifugation and concentration, the aqueous solution was cleaned up on a Chromabond XTR™ column. Residues were eluted with 40 mL of dichlormethane, dried and collected in 2 mL of toluene/ethyl acetate (85/15 v/v). After the addition of 5 mL of acetonitrile and 5 mL of toluene/ethyl acetate (85/15 v/v), the solution was passed through a 0.5 mg Silica Gel™ column. Residues were rinsed with toluene ethyl acetate (70/30 v/v), passed through the column again, eluted with acetonitrile/water (95/5 v/v), dried and dissolved in 1 mL of methanol/water (1/9 v/v) and subjected to HPLC‐MS/MS.

### 2.7 Parameters of colony performance

As parameters of colony strength, the number of brood cells and the number of adult bees per comb were visually estimated for each colony according to the Liebefeld method.^20^ Colony strength was assessed 8 times per replication, 4 times before and 4 times after overwintering. The individual assessments per season (autumn/spring) were performed at intervals of 21 days (± 1 day). On each assessment date, the weight of the colonies was also determined using a digital balance. Box‐shaped wire grids with a mesh size of 8 mm were mounted in front of the entrances to collect dead bees and measure mortality. During the periods of bee flight, traps were emptied twice a week and the number of dead bees recorded. Prior to the first cleansing flight in early spring, dead bees from the bottom boards of the hives were collected, weighed and counted to estimate the number of bees that had died during winter. Winter colony losses and queen losses were recorded during regular inspections of the colonies.

### 2.8 Statistical evaluation

Statistical analyses were conducted with the software package SPSS v.20. Weights, numbers of bees and numbers of brood were checked for normality with the Shapiro–Wilks test, and for homogeneity of variances by Levene's test. Where required, data were subjected to transformation (square root or other as indicated in the respective figures) and analysed with a repeated‐measures ANOVA according to the glm repeated statement,^21^ where group and year were considered as between‐subject factors. In the case of the brood values, no adequate transformation of the data was found. Nevertheless, repeated‐measures ANOVA was conducted, as the group sizes were nearly equal, and therefore violations of assumptions of data normality and variance homogeneity were considered not to affect the analysis.^22^ The assumption of sphericity was tested with Mauchly's test. In the case of violation of the sphericity assumption, the degrees of freedom were adjusted using the box correction (in SPSS denoted as the Greenhouse Geisser correction)^21^ or by the Huynh and Feldt correction (if *ϵ* ≥ 0.75). If a significant overall effect was found, groups were compared against each other with Tukey's test (if *ϵ* ≥ 0.75; otherwise the Bonferroni procedure was used).^23^ To compare the parameter ‘number of dead bees from the dead bee traps’, the area under the curves (AUC) was calculated and tested for normality. Where required, the values were log transformed and subjected to ANOVA (proc univariate) in order to test the differences between groups. The number of dead bees from the bottom board of the hives in winter was square root transformed and subjected to ANOVA (proc univariate). For the parameter ‘number of bees’, a power analysis was conducted with the software g*power.^24^ To this end, the correlations between the measurements were *Z* transformed according to Rasch.^25^ The required sample size for a medium effect with an effect size *f* = 0.25 was estimated with the g*power inherent procedure ‘ANOVA, repeated measures’. As the data of *Nosema* spp. had a skewed distribution with multiple zero values, the non‐parametric Kruskal–Wallis test was used for comparisons.^26^

The *C*_t_ values of the real‐time PCR analyses for ABPV and the hymenoptaecin gene expression analyses were evaluated using the $2^{-\Delta \Delta C_{t}}$ method relative to the controls of the first year.^27^ Outliers within groups were identified and eliminated by Grubb's test (two within the ABPV values and four within the rp49 values). Data were log (*x* + 1) transformed to approximate variance homogeneity. Means of the groups expressed as the fold increase in ABPV loads relative to the control group of year 1 were compared with the aid of glm univariate in SPSS. For the gene expression experiment, no adequate data transformations to approximate normal distribution and homogeneity of variances were found. Therefore, outliers were identified by exploring the data as box plots in SPSS. Datasets in which rp49 values were above the 75 percentile or below the 25 percentile were eliminated, as the outlying rp49 results were interpreted as failures in the extraction process of RNA. Kruskal–Wallis tests were performed to check for significant differences between the injected and the non‐injected groups. In the case of multiple comparisons, *α*‐values were corrected according to Bonferroni.

## 3 RESULTS

### 3.1 Pathological characterisation of the colonies

As the colonies were carefully treated for *Varroa* with coumaphos, the observed rates of mite infestation were low. The means averaged over all three years were 0.0316 mites g^−1^ bees for the C colonies (SD = 0.0453; *N* = 90), 0.0332 mites g^−1^ bees for the T1 colonies (SD = 0.0543; *N* = 90) and 0.0395 mite g^−1^ bee for the T2 colonies (SD = 0.0749; *N* = 90). There were no significant effects of thiacloprid on the level of *Varroa* infestation (*P*_THIA_ = 0.412; *P*_year_ < 0.001). At the beginning of August the level of infestation ranged from 0 to 0.0581 mites g^−1^ bee. In mid‐September the minimum was 0 and the maximum was 0.1691 mites g^−1^ bee, and in October the range was 0–0.4621 mites g^−1^ bee. After the application of coumaphos in winter, 303.5 mites on average dropped from the controls (SD = 254, *N* = 30). From the T1 colonies, 283.1 mites dropped on average (SD = 245.9; *N* = 30), and from the T2 colonies 261.8 mites dropped on average (SD = 187; *N* = 30). The number of dropped mites did not differ significantly between groups (*P* = 0.698, proc glm univariate), but was different for the factor ‘year’ (*P* < 0.001, proc glm univariate). The colonies were tested for viral infections. The PCRs of all samples were negative for chronic bee paralyis virus (CBPV). Deformed wing virus (DWV) was rare, with nine positives out of 84 samples detected: two in controls, two in T1 colonies and five in T2 colonies. In contrast, acute bee paralysis virus (ABPV) was frequently detected. The factor ‘treatment’ had no significant effect on the relative ABPV load (*P* = 0.619). Differences between years were significant (*P* = 0.003). The fold values of ABPV were 2.5 for C (*N* = 9), 16.1 for T1 (*N* = 10) and 8.1 for T2 (*N* = 10) in year 1, 95.0 for C (*N* = 5), 55.9 for T1 (*N* = 8) and 2.1 for T2 (*N* = 9) in year 2 and 56.0 for C (*N* = 9), 47.6 for T1 (*N* = 10) and 77.7 for T2 (*N* = 8) for year 3. Spores of *Nosema* spp. were detected, especially in the samples collected during summer. Control bees from the end of July/beginning of August contained 2.4 × 10^6^ spores bee^−1^ (SD = 2.8 × 10^6^, *N* = 30) versus 2.0 × 10^6^ spores bee^−1^ (T1, SD = 2.1 × 10^6^, *N* = 30) and 2.1 × 10^6^ spores bee^−1^ (T2, SD = 3.2 × 10^6^, *N* = 30). Differences were not significant (*P* = 0.761, Kruskal–Wallis test). The loads of *Nosema* spp. in autumn and spring were lower. The respective mean values for autumn were 0.10 × 10^6^, 0.10 × 10^6^ and 0.11 × 10^6^ (*P* = 0.404, Kruskal–Wallis test) and for spring 0.3 × 10^6^, 0.0 × 10^6^ and 0.1 × 10^6^ spores bee^−1^ (*P* = 0.068, Kruskal–Wallis test).

### 3.2 Performance and strength of colonies

**Figure 1:**
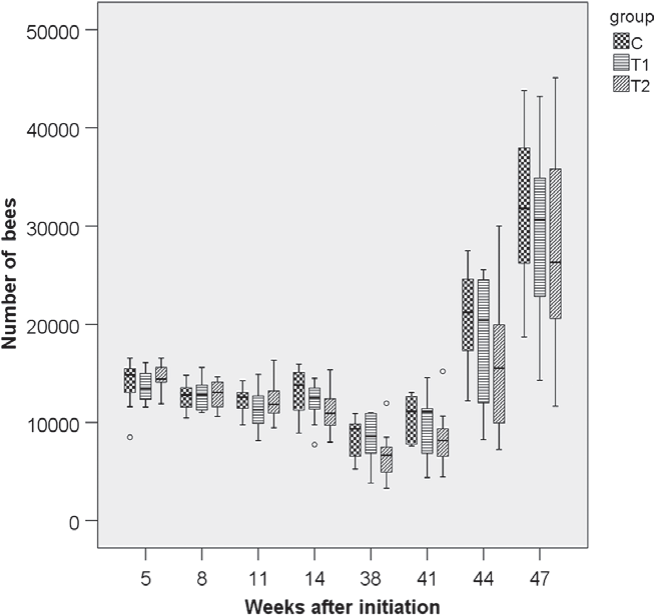
Number of adult bees in 2011/2012 for the three groups. N = 10 colonies per group; for bees: P_treatment_ = 0.113, P_year_ < 0.001 (glm repeated measurements). C: control colonies, not exposed to thiacloprid; T1: colonies fed with syrup spiked with 0.2 mg thiacloprid L^−1^; T2: colonies fed with syrup spiked with 2 mg thiacloprid L^−1^.

**Figure 2:**
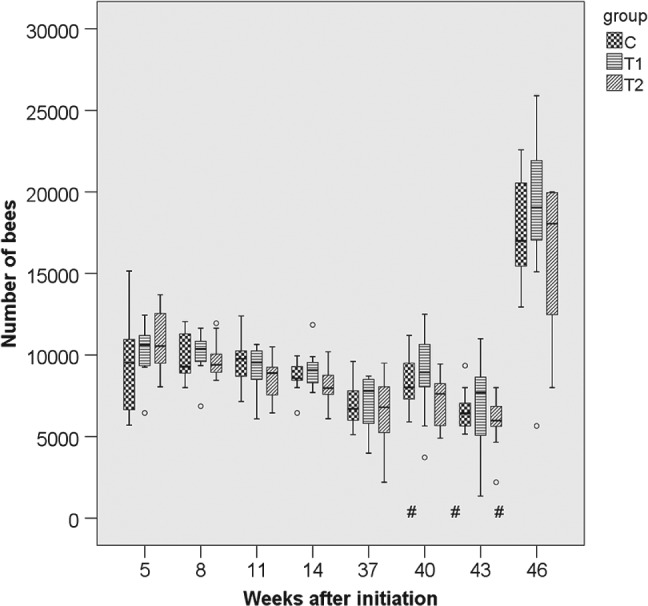
Number of adult bees in 2012/2013 for the three groups. N = 10 colonies per group; for bees: P_treatment_ = 0.113, P_year_ < 0.001 (glm repeated measurements). C: control colonies, not exposed to thiacloprid; T1: colonies fed with syrup spiked with 0.2 mg thiacloprid L^−1^; T2: colonies fed with syrup spiked with 2 mg thiacloprid L^−1^; boxes labelled with ‘#’: only nine colonies of group T2 could be evaluated (one irreplaceable failure).

**Figure 3:**
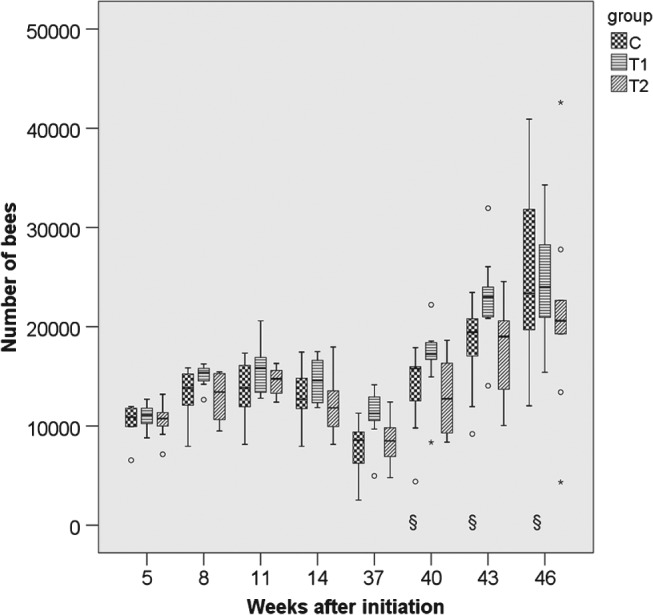
Number of adult bees in 2013/2014 for the three groups. N = 10 colonies per group; for bees: P_treatment_ = 0.113, P_year_ < 0.001 (glm repeated measurements). C: control colonies, not exposed to thiacloprid; T1: colonies fed with syrup spiked with 0.2 mg thiacloprid L^−1^; T2: colonies fed with syrup spiked with 2 mg thiacloprid L^−1^; boxes labelled with ‘§’: only nine colonies of group C could be evaluated (one irreplaceable failure).

**Figure 4:**
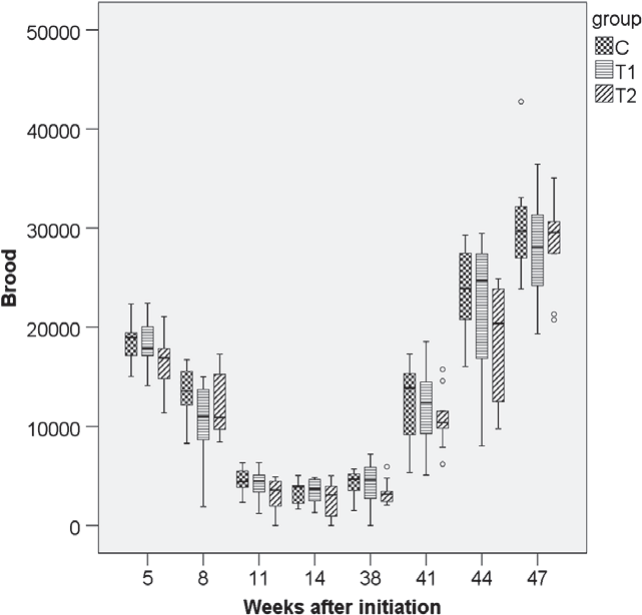
Number of brood cells in 2011/2012 for the three groups. N = 10 colonies per group; for bees: P_treatment_ = 0.037, P_year_ < 0.001 (glm repeated measurements). C: control colonies, not exposed to thiacloprid; T1: colonies fed with syrup spiked with 0.2 mg thiacloprid L^−1^; T2: colonies fed with syrup spiked with 2 mg thiacloprid L^−1^.

**Figure 5:**
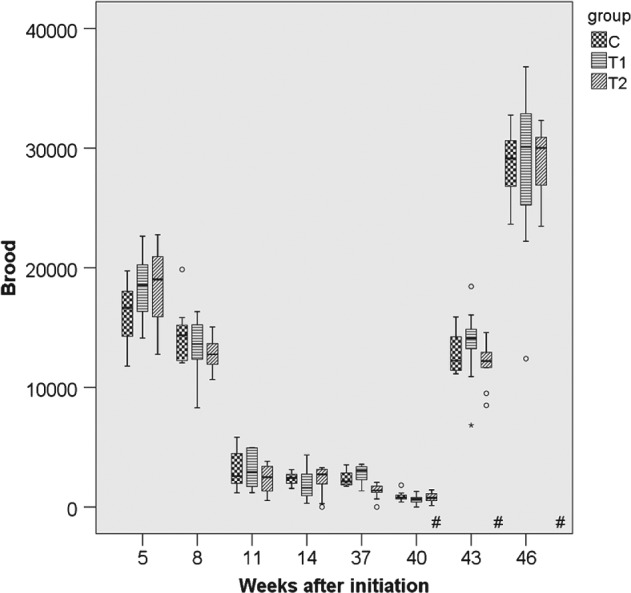
Number of brood cells in 2012/2013 for the three groups. N = 10 colonies per group; for bees: P_treatment_ = 0.037, P_year_ < 0.001 (glm repeated measurements). C: control colonies, not exposed to thiacloprid; T1: colonies fed with syrup spiked with 0.2 mg thiacloprid L^−1^; T2: colonies fed with syrup spiked with 2 mg thiacloprid L^−1^; boxes labelled with ‘#’: only nine colonies of group T2 could be evaluated (one irreplaceable failure).

**Figure 6:**
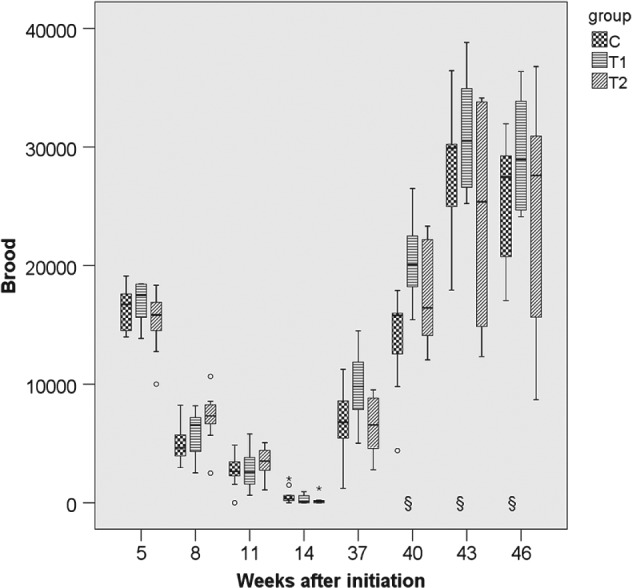
Number of brood cells in 2013/2014 for the three groups. N = 10 colonies per group; for bees: P_treatment_ = 0.037, P_year_ < 0.001 (glm repeated measurements). C: control colonies, not exposed to thiacloprid; T1: colonies fed with syrup spiked with 0.2 mg thiacloprid L^−1^; T2: colonies fed with syrup spiked with 2 mg thiacloprid L^−1^; boxes labelled with ‘§’: only nine colonies of group C could be evaluated (one irreplaceable failure).

The parameters ‘colony strength’ (number of adult bees), ‘number of brood cells’ and ‘weight gain’ were recorded for 10 months per study replication. For the parameter ‘adult bees’ the differences between groups were small and not statistically significant (*P*_THIA_ = 0.113; *P*_year_ < 0.001) (Figs 1, 2, 3, 4, 5, 6). With respect to brood, significant differences were found (*P*_THIA_ = 0.037; *P*_year_ < 0.001). The numbers of brood cells of the C colonies and of the T2 colonies were similar. The numbers of brood cells of the T1 colonies were statistically significantly different from the T2 colonies (*P* = 0.032, Bonferroni procedure) but not from the controls (*P* = 0.811, Bonferroni procedure). Interactions between time of measurement and treatment were not significant for the number of adult bees (*P* = 0.125, box correction) or for the number of brood cells (*P* = 0.061, box correction). The values averaged over all eight estimation dates and all three years are shown in Table 1. In spring, colonies started the production of drone brood. Drone brood was recorded at four time points. Differences between groups were not significant (*P* = 0.169; values were square root (*x* + 1) transformed). However, the year was a significant factor (*P* < 0.001). Interactions between the time of the measurements and the exposure to thiacloprid were not significant (*P* = 0.127, Huynh–Feldt correction). The net weights of the colonies were similar, irrespective of exposure to thiacloprid (*P*_treatmenat_ = 0.166; *P*_year_ < 0.001). Mean net weights of 37.17 kg (*N* = 237; SE = 0.612), 37.96 kg (*N* = 240; SE = 0.679) and 36.40 kg (*N* = 238; SE = 0.609) were observed for the control, T1 and T2 treatments respectively. Interaction between time of measurement and treatment were significant (*P* = 0.009, Huynh–Feldt correction).

**Table 1:** Number of bees, worker brood cells and drone brood cells. Shown are means, SE and N of all three years of the experiment. Groups marked with different letters are significantly different (Tukey's test)

	Number of bees			Number of worker brood cells			Number of drone brood cells		
	Mean	SE	*N*	Mean	SE	*N*	Mean	SE	*N*
0 mg THIA L^−1^	13 151 a	448	237	11 918 ab	636	237	3121 a	302	116
0.2 mg THIA L^−1^	13 645 a	421	240	12 571 b	670	240	2846 a	281	120
2 mg THIA L^−1^	12 277 a	412	237	11 344 a	623	237	2555 a	272	116

### 3.3 Estimation of the required sample sizes for detecting medium effects

**Figure 7:**
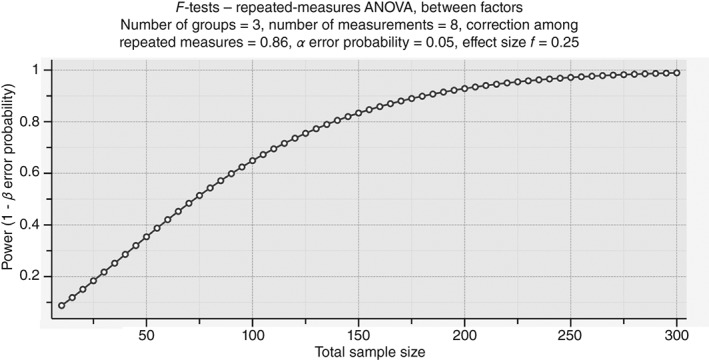
Total number of samples that are required to measure a medium effect of f = 0.25 for the parameter ‘number of bees’ with α = 0.05 and β = 0.80. Power analysis performed with g*power. The total sample was calculated.

For the parameter ‘number of adult bees’, a high correlation between repeated measures with *ρ* = 0.86 (mean Pearson correlation coefficient) was found. Using the g*power inherent procedure ‘ANOVA: repeated measures’, we calculated the total sample size required for detecting a medium effect of *f* = 0.25 with a power of 1 − *β* error probability of 0.8 and an *α* error probability of 0.05 (Fig. 7). The total sample size amounted to 141 colonies. In the actual study design with three groups and three years, 15.7 colonies are sufficient to verify a medium effect with an adequate power of 0.8.

### 3.4 Mortality of bees

Adult bee mortality in dead bee traps was regularly counted. In total, 1920 measurements were recorded in 2011/2012, 1726 measurements in 2012/2013 and 1937 measurements in 2013/2014. After aggregation by the AUC approach, 90 values were obtained for analysis of variance. The effect of the exposure of thiacloprid on the number of dead bees in dead bee traps was not significant (*P* = 0.728, data log transformed). The effect of the factor ‘year’ was highly significant (*P* < 0.0001). Interactions were not significant (*P* = 0.143). The respective means are shown in Table 2. At the end of each winter, shortly before the beginning of flight activity, the dead bees on the bottom boards of the hives were collected and counted. On average, C colonies had 1027 dead bees per colony (SD = 744; *N* = 30), T1 colonies had 742 dead bees per colony (SD = 623; *N* = 30) and T2 colonies had 719 dead bees per colony (SD = 668; *N* = 30). Differences between groups and years were significant (*P*_year_ < 0.001, *P*_treatment_ < 0.001; data square root transformed, interaction n.s., *P* = 0.107).

**Table 2:** Number of dead bees collected in the traps fixed in front of the entrances

	Mean year 1	Mean year 2	Mean year 3	Overall mean
0 mg THIA L^−1^	26.55	21.28	11.69	19.84
0.2 mg THIA L^−1^	25.90	25.02	10.22	20.38
2 mg THIA L^−1^	25.13	22.15	12.01	19.76

### 3.5 Queen failure and colony survival

The exposure to thiacloprid had no effect on overwintering success. None of the colonies failed during winter. In each year, one queen out of the ten C colonies and one queen out of the ten T2 colonies were lost. In 2011/2012 and 2012/2013, two queens of the ten T1 colonies failed. In the first year, all colonies survived until the end of the observation period. Spring failures occurred in 2013 (one T2 colony) and in 2014 (one C colony). Neither losses were replaced.

### 3.6 Residues of thiacloprid

Thiacloprid and its metabolite thiacloprid‐amide were detected in syrup and in bee bread stored in the combs of the hives. In autumn, control colonies contained stored syrup with ∼10 µg thiacloprid kg^−1^, and treated colonies T1 ∼ 150 µg kg^−1^ and T2 ∼ 780 µg kg^−1^ (Table 3). Before winter, the concentrations of thiacloprid in the investigated matrices were slightly higher than after winter. In spring, residues were 12 µg kg^−1^ in C colonies, 94 µg kg^−1^ in T1 colonies and 631 µg kg^−1^ in T2 colonies, which demonstrates that the colonies were in fact exposed the whole time from the beginning of the experiment in July until spring. In autumn, the concentrations of thiacloprid‐amide ranged between 0.3 µg kg^−1^ (C) and ∼8 µg kg^−1^ (T2) in stored syrup. In the treated colonies, bee bread contained lower residue levels of thiacloprid and thiacloprid‐amide compared with the stored syrup. In spring, the concentrations in bee bread were similar to the values observed in autumn.

**Table 3:** Residues of thiacloprid (THIA) and thiacloprid‐amide (in µg kg^−1^) found in stored syrup and bee bread of C colonies, of T1 colonies and of T2 colonies. Given are the means, minimum and maximum and N values calculated over all three years^a^

Group		Autumn		Spring		Autumn		Spring	
		Stored food				Bee bread			
		THIA	THIA‐amide	THIA	THIA‐amide	THIA	THIA‐amide	THIA	THIA‐amide
C, 0 mg THIA L^−1^	mean	10.19	1.18	11.59	5.56	20.40	1.48	7.25	0.99
	min	<LOQ	<LOQ	<LOQ	<LOQ	<LOQ	<LOQ	<LOQ	<LOQ
	max	48.00	2.26	65.62	13.10	304.21	3.70	44.40	1.31
	*N*_quant_	26	2	28	3	23	4	28	4
	*N*_<LOQ_	4	28	2	27	7	26	2	26
T1, 0.2 mg THIA L^−1^	mean	156.11	1.53	93.96	222	45.71	1.65	44.51	1.71
	min	33.81	<LOQ	1.36	<LOQ	9.64	<LOQ	8.50	<LOQ
	max	1318.30	2.33	187.40	4.60	274.03	3.17	331.60	7.40
	*N*_quant_	30	29	30	29	30	4	30	22
	*N*_<LOQ_	0	1	0	1	0	26	0	8
T2, 2 mg THIA L^−1^	mean	777.76	7.77	630.57	13.42	203.28	615	205.89	4.36
	min	473.83	1.30	172.10	<LOQ	1.24	<LOQ	40.14	1.63
	max	1278.20	12.44	882.40	21.10	369.1	14.65	441.00	12.78
	*N*_quant_	30	30	29	28	28	26	29	29
	*N*_<LOQ_	0	0	0	1	0	2	0	0

a LOD = 0.0001 mg kg^−1^; LOQ = 0.001 mg kg^−1^.

### 3.7 Expression of the hymenoptaecin gene

**Figure 8:**
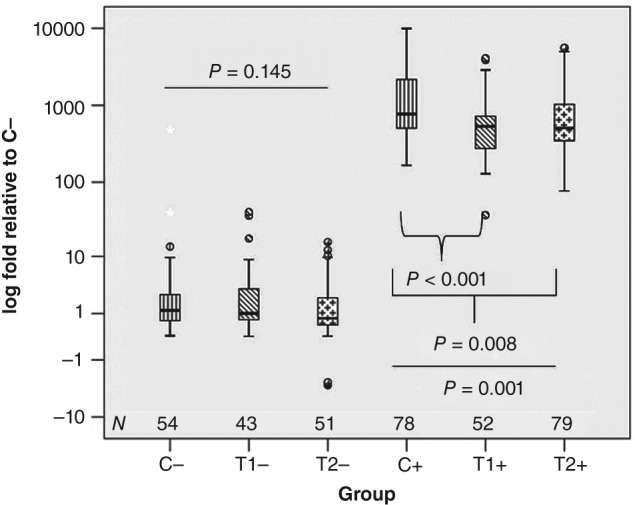
Expression of the hymenoptaecin gene relative to the controls of the first year without injection of P. larvae: on the left side, naive bees without injection of P. larvae (marked with ‘−’); on the right side, bees that were challenged with P. larvae (marked with ‘+’). Kruskal–Wallis test, P_with injection_ < 0.001; P_without injection_ = 0.145; multiple testing C+ versus T1+ and C+ versus T2+, α = 0.025, Bonferroni corrected. N is the number of tested bees.

The constitutive expression and the challenged amount of mRNA of the hymenoptaecin gene were measured. Exposure to thiacloprid did not change the constitutive expression. However, after injection of *P. larvae* suspensions, bees exposed to thiacloprid expressed significantly lower levels of hymenoptaecin mRNA than bees of the control groups (Fig. 8).

## 4 DISCUSSION

Two groups of ten colonies each were exposed to different concentrations of thiacloprid by means of in‐hive feeding of thiacloprid‐spiked sugar syrup. Two concentrations, 0.2 and 2 mg thiacloprid L^−1^, were administered. Control colonies were fed with syrup containing the solvent acetone alone. The study simulated a long‐term exposure with sublethal thiacloprid concentrations. The test was replicated 3 times in three subsequent years.

The dosages were chosen so as to mimic a realistic field situation (T1 a lower‐level exposure group) or an unrealistically exaggerated exposure (T2 a higher‐level exposure group). Thiacloprid has been found in bee‐collected pollen.^4^ Pollen collected by foragers from treated apple flowers contained 0.03 mg thiacloprid residues kg^−1^.^28^ Peak values of thiacloprid residues are reported from a Germany‐wide survey (the German Bee Monitoring). A maximun concentration of 498 µg kg^−1^ was found in bee bread in 2012.^29^ Concentrations in Austrian honeys varied widely between values below LOD and values up to 27.4 µg kg^−1^.^5^ Oilseed rape flowers treated with 50 g thiacloprid ha^−1^ (PROTEUS 110 O™) had on average 6.5 µg thiacloprid kg^−1^ in nectar (maximum 208.8 µg kg^−1^ and median 2.5 µg kg^−1^; 64% positive samples) and 89.1 µg thiacloprid kg^−1^ in pollen (maximum 1002.2 µg kg^−1^ and median 4.1 µg kg^−1^; 62% positive samples).^30^ Bee‐collected nectar and honey contained around 2 µg thiacloprid kg^−1^, whereas residues in bee‐collected pollen and bee bread reached mean levels of up to 81.6 µg thiacloprid kg^−1^.^30^ A study from the United States reports much lower concentrations in pollen (mean 23.8 µg kg^−1^; ranging from 1.7 to 115 µg kg^−1^, with only 5.4% positive samples out of 350).^31^ Likewise, a recent Dutch study reports lower frequencies and lower residue levels in bee‐collected matrices (bee‐collected pollen <66 µg kg^−1^; honey <15 mg kg^−1^).^32^ The heterogeneity of these findings can be explained by the different study designs, such as whether matrices of directly treated crops or of variable sources were analysed, and by the different patterns of usage and thiacloprid‐treated crops in different countries. There are no reports that describe findings of residue levels as high as 2 mg thiacloprid kg^−1^ in bee‐relevant matrices under realistic exposure scenarios. The extremely long‐lasting exposure over winter is not a field‐relevant scenario. Under practical conditions, beekeepers remove the honey in summer and feed the colonies with sugar syrup which is subsequently consumed during winter. Consequently, the high concentration of 2 mg L^−1^ and the extremely long duration of exposure of our study represent an unrealistic worst‐ case scenario.

The parameters ‘colony strength’, ‘number of brood cells’, ‘production of drones’, ‘hive weight gain’, ‘overwintering success’ and ‘mortality’ were chosen as endpoints. According to the EFSA,^33^ colony strength is by far the most important and the most meaningful endpoint. In our experiment, no significant impact of the treatment on colony strength was observed. As the interaction between the factors ‘time’ and ‘exposure to thiacloprid’ was not significant, there was also no delayed effect of thiacloprid on colony strength. Although the administered concentrations of thiacloprid were below the lethal level, a reduction in colony strength through sublethal effects might be expected, such as a reduction in the lifespan of the bees owing to the long duration of exposure. Foragers leaving the hive supposedly consume thiacloprid containing syrup before departing. Several studies report effects of neonicotinoids on the orientation, foraging and homing capacity of honey bees,^8^, ^34^, ^35^, ^36^ and several other sublethal effects of neonicotinoids are postulated that could lead to a depopulation of the colony even months after exposure.^37^ The data on colony strength from our study do not support these hypotheses.

Perhaps even more important than the statistical significance of an experiment is the magnitude of the effects found.^38^ According to the EFSA,^33^ a reduction in colony strength of 7% can be considered negligible. Colonies of the T1 group were the strongest in absolute numbers of bees, averaged over all three years. A reduction in the mean strength of the control colonies by 7% would correspond to a colony strength of 12 230 bees. The mean number of T2 colonies with 12 277 bees was still within that range. Against this background, a potential effect of the thiacloprid treatment appears to be of insignificant magnitude.

Regarding the endpoint ‘brood’, differences between C colonies and the T1 colonies were not significant. However, the T2 colonies raised significantly less brood than the T1 colonies. The reduction in brood of T2 colonies was consistent over all three years. However, the brood cell numbers of the T2 colonies were not statistically significantly different from the control colonies. The respective values for T2 were 11 969 brood cells in year 1, 9699 in year 2 and 12 312 in year 3 versus 12 851, 10 259 and 14 604 for the T1 colonies. Nevertheless, it has to be noted that the reduction in brood did not result in any significant differences in colony strengths. To date there have been no reports of a direct brood toxicity of thiacloprid, but for other neonicotinoids brood effects such as delayed development have been reported.^4^ Indirect effects could also play a role, such as reduced olfactory capacities or constrained locomotory activity of nurse bees that could result in suboptimal brood temperature. The causality and the mechanisms behind the differences in brood production remain unclear. Here, too, interactions between time and exposure to thiacloprid were not significant, and thus the data do not support the hypothesis of a delayed impact on the production of brood. For thiamethoxam and clothianidin, Sandrock *et al*.^39^ found a long‐term reduction in the number of bees and of brood cells, possibly linked to the performance of the queens. In the case of thiacloprid, detrimental effects appearing a long time after the exposure were not confirmed.

The endpoint weight development of the colonies was not affected by thiacloprid. All colonies were able to take up and store the sugar syrup, draw out the wax foundation and build up normal colonies of similar weights. The weight data did not provide any indication of impaired functionality of the colonies.

We did not observe increased mortality of colonies in winter, reduced survival of the queens or impairment of the production of drone brood in spring. The reproductive capacity of the treated colonies in comparison with the control colonies was not affected. These observations do not support the conclusion of colony level effects of thiacloprid as derived from the study of van der Zee *et al*.:^32^ this observational pilot study identified thiacloprid residues in honey and in bees as a significant risk factor for colony collapse in winter. However, it remains unclear in this study whether the association between higher relative risk for overwinter survival and the presence of neonicotinoids was coincidental or causal. Other authors have reported strong impacts of the neonicotinoids thiamethoxam, clothianidin and imidacloprid on queen production and queen survival in *Apis mellifera* 40 and *Bombus* sp.^41^, ^42^, ^43^, ^44^ and on drone production in *A. mellifera*.^45^ We did not observe similar effects of thiacloprid.

There was no increased acute mortality of adult bees in the thiacloprid‐exposed colonies. During the exposure period, the number of dead bees in the dead bee traps was similar in the treatment and the control colonies. However, the number of dead bees collected at the end of winter in the bottom boards did differ significantly between the three groups, with the highest numbers of dead winter bees observed in the controls. As thiacloprid has a moderate acute toxicity to bees, an elevated mortality concurring with the oral exposure could not be expected; interestingly, however, even long‐lasting exposure during autumn and winter did not result in increased levels of mortality, either of individual bees or of colonies. As shown by the residue levels in the stored syrup before and after winter, the bees were in fact continuously exposed. Even though the content of thiacloprid in the body of the exposed bees was not measured in this study, the mortality data show that the bees were apparently able to prevent an accumulation of thiacloprid to toxic levels. The underlying mechanism is not yet fully understood. A metabolisation of thiacloprid by way of the P450 detoxification system, by oxidisation processes or by excretion has been discussed.^3^, ^46^, ^47^ In this context it is worth mentioning that the colonies were treated against Varroosis with coumaphos, and thereby had to cope with the double stress of simultaneous application of both synthetic drugs. Apparently, the body burden on the bees was such that they were able to tolerate it, which is in agreement with the literature. Synergisms between certain neonicotinoids and other pesticides are documented, but coumaphos and thiacloprid have been reported not to interact.^48^, ^49^

The concentrations of thiacloprid used in our experiment corresponded to a worst‐case field‐realistic scenario (T1) and a tenfold increased unrealistic worst‐case scenario (T2). Beyond that, we did not include additional extreme concentrations to enforce effects of an overdose of thiacloprid on honey bee colonies. While this could be seen as critical, there are, in fact, no operating procedures known to us that instruct to include positive controls in field assays with the goal to show the assay's sensitivity. Accordingly, no toxic standards are defined or validated for field study designs. Toxic standards are not considered feasible in field trials, and positive controls are therefore not used, especially when the exposure can be shown by other means such as residue analysis, as done in our study, or observation of foraging activity in a treated crop. In fact, current testing guidelines do not recommend positive controls in field assays of pesticide testing.^50^, ^51^

The EFSA requested that, in pesticide testing, field assays should generate data that can statistically be evaluated with a meaningful power.^52^ Therefore, we estimated the sample size that is required for detecting a statistically significant medium effect (Fig. 7). Approximately 15 colonies per group and year were sufficient. Such rather high numbers of replicates raise the question of practicability, which should be considered in defining a regulatory study design.^53^ Other guidelines state that the options for a statistical evaluation of field test data are limited. Limitation of statistical power is due to the relatively low number of replicates that are feasible and the possible violation of the independence assumption.^51^ The guidelines conclude that descriptive rather than interfering statistics might be appropriate. It was intended to overcome these limitations by the described study design of free‐ranging colonies exposed by in‐hive feeding, as it allows handling a larger number of replicates. In our study, ten colonies per treatment and year were each assessed 8 times, resulting in a calculated total of 720 datasets (effectively 714 datasets, as six datasets were not recordable owing to colony failure). Despite the extensive number of datasets, the power would not have satisfied the criteria according to the EFSA.^33^ Further research on the improvement of the testing scheme may be helpful to overcome this issue.

In our experiment, the colonies were placed together in groups, and not distributed through the apiary following a random pattern. While it is debatable whether this arrangement can truly be considered to provide independent replicates, this grouping was favoured over a random pattern with the objective of minimising the risk of carryover of residues during colony handling and of minimising worker drifting between colonies from different experimental groups.^54^ Drifting was probably not completely prevented, as low amounts of thiacloprid residues were found even in the stores of control colonies. Similar pesticide shifts to the controls by drifting bees or from the outside were observed by others as well,^55^ but nevertheless the studies are meaningful, as the residues increased substantially in the expected directions.

In every field test, a balance has to be made between having all treatment units exposed to the same environmental factors, filtering out an important part of natural variability and excluding potential influences between the different testing units. In our study we chose an approach that places particular emphasis on the exclusion of the influence of variable environmental factors by setting up all treatment groups together in one structurally homogeneous location. For instance, there were no trees that shaded a few colonies only, or any other evident environmental factors differentially influencing one of the groups. An alternative approach would have been to set up all colonies individually, in order to exclude interactions between the individual units. This would in turn have had the disadvantage that a higher number of replicate colonies would have been needed in order to compensate for the variability brought in by the differences between the different sites. In reality, however, owing to the increased time needed for the handling of the hives in many different locations, fewer replicates would have been possible for logistical reasons. Overall, we consider the approach of exposure by in‐hive feeding and placing ten colonies per group in a homogeneous test location a valuable design. It allows a controlled exposure and a statistical evaluation, even if small effects may not have been detectable as a result of the relatively low statistical power of the system. It has been suggested that power could be improved by increasing the number of replicates, which is, however, extremely difficult in practice for reasons of experiment logistics, and/or by reducing the intrinsic variation, e.g. with the help of better estimation methods.^56^ Future studies should address this latter aspect.

Pesticides can exert immunosuppressive effects.^57^ There are documented cases of immunotoxicity in insects.^58^, ^59^ So far, little is known for honey bees. The exposure to multiple xenobiotics confronting honey bees in the environment has been discussed as a contributing factor to colony losses.^60^ It is so far not well understood whether neonicotinoids can affect the functionality of the immune system of honey bees under practical field conditions. Clothianidin has been reported as an immunosuppressive agent rendering bees more susceptible to viral diseases.^61^ Other studies suggest that neonicotinoids increase the susceptibility to *Nosema* spp.,^55^, ^62^, ^63^ which has also been reported for thiacloprid.^10^, ^11^ Such effects have, however, so far not been observed under realistic field conditions in the context of entire bee colonies. The significance of the presumed interactions between pathogens and pesticides is therefore still under discussion. A recent study concludes that the interactions might be overemphasised.^64^ According to Brandt *et al*.,^65^ exposure of bees to thiacloprid can modify their numbers of haemocytes and their capacity to nodulate foreign objects. Haemocytes and nodulation are considered important constituents of the immune system of insects.^66^ The study reported here compared bees from thiacloprid‐exposed colonies with control bees. As an immunity‐related parameter, the expression of the gene encoding for hymenoptaecin was measured. This gene encodes the production of an antimicrobial peptide, which is effective in defending against bacteria.^67^ It is easily quantifiable, as the regulation of its expression can change by up to two orders of magnitude.^68^ The thiacloprid‐exposed bees showed a slight but statistically significant modification in the expression pattern. They produced less hymenoptaecin mRNA than control bees. The magnitude of the effect was low, however, and we did not observe any impact on the colonies that may have been caused by this modification. The colonies were in good health. The parasitisation rates of *V. destructor* were low in all treatment groups, and *Nosema* spp. infestations were within normal ranges. The loads of viral pathogens were without pathologically abnormal findings. As the colonies were initiated from treated, shook swarms on virgin wax foundation with young queens, health problems were in fact unlikely. The pathogen burdens of control and treated colonies were of a similar low level. Therefore, it is not possible to deduce from the findings of this study a biological significance of immunomodifying effects of thiacloprid at the colony level. Further research is required, focusing on colonies suffering from pathogen and environmental stress, which is believed to aggravate the effect of pesticides.^55^, ^61^, ^62^

We have demonstrated that healthy and well‐managed honey bee colonies are not adversely affected by a chronic exposure to thiacloprid at realistic and even at unrealistically exaggerated exposure levels. No adverse effects on colony strength, overwintering capacity or performance of queens were observed. Our study did not address the topic of potential synergisms between different pesticides or the performance of thiacloprid‐exposed colonies under poor health conditions. Future research could deepen our knowledge about these topics.

## References

[1] *Absatz an Pflanzenschutzmitteln in der Bundesrepublik Deutschland* . Ergebnisse der Meldungen gemäß 64 Pflanzenschutzgesetz für das Jahr 2014. [Online]. Bundesamt für Verbraucherschutz und Lebensmittelsicherheit (2015). Available: http://www.bvl.bund.de/psmstatistiken [23 March 2016].

[2] Schmuck R , Ecotoxicological profile of the insecticide thiacloprid. Pflschutz Nachr. Bayer Engl Edn 54:161–184 (2001).

[3] Iwasa T , Motoyama N , Ambrose JT and Roe RM , Mechanism for the differential toxicity of neonicotinoid insecticides in the honey bee, Apis mellifera. Crop Prot 23:371–378 (2004).

[4] Blacquiere T , Smagghe G , Van Gestel CA and Mommaerts V , Neonicotinoids in bees: a review on concentrations, side‐effects and risk assessment. Ecotoxicology 21:973–992 (2012). 22350105 10.1007/s10646-012-0863-x PMC3338325

[5] Tanner G and Czerwenka C , LC‐MS/MS analysis of neonicotinoid insecticides in honey: methodology and residue findings in Austrian honeys. J Agric Food Chem 59:12 271–12 277 (2011). 10.1021/jf202775m 22026460

[6] Genersch E , Von der Ohe W , Kaatz H , Schroeder A , Otten C , Büchler R *et al*, The German bee monitoring project: a long term study to understand periodically high winter losses of honey bee colonies. Apidologie 41:332–352 (2010).

[7] Von der Ohe W and Martens D , Das Deutsche Bienenmonitoring, Pflanzenschutzmittel‐Rückstände im Bienenbrot. ADIZ/db/IF 10:8–9 (2011).

[8] Fischer J , Müller T , Spatz A‐K , Greggers U , Gruenewald B and Menzel R , Neonicotinoids interfere with specific components of navigation in honeybees. PLoS ONE 9:e91364 (2014). 10.1371/journal.pone.0091364 PMC3960126 24646521

[9] Laurino D , Porporato M , Patetta A and Manino A , Toxicity of neonicotinoid insecticides to honey bees: laboratory tests. Bull Insectol 64:107–113 (2011).

[10] Vidau C , Diogon M , Aufauvre J , Fontbonne R , Viguès B , Brunet J‐L *et al*, Exposure to sublethal doses of fipronil and thiacloprid highly increases mortality of honeybees previously infected by *Nosema ceranae* . PLoS ONE 6:e21550 (2011). 10.1371/journal.pone.0021550 PMC3125288 21738706

[11] Doublet V , Labarussias M , de Miranda JR , Moritz RF and Paxton RJ , Bees under stress: sublethal doses of a neonicotinoid pesticide and pathogens interact to elevate honey bee mortality across the life cycle. Environ Microbiol 17:969–983 (2015). 25611325 10.1111/1462-2920.12426

[12] Dietemann V , Nazzi F , Martin SJ , Anderson DL , Locke B , Delaplane KS *et al*, Standard methods for varroa research. J Apic Res 52:1–54 (2013).

[13] Nosemosis of honey bees . OIE Terrestrial Manual. [Online]. Office International des Epizooties (OIE) (2013). Available: http://www.oie.int/fileadmin/Home/eng/Health_standards/tahm/2.02.04_NOSEMOSIS_FINAL.pdf [18 February 2016]

[14] Siede R , König M , Büchler R , Failing K and Thiel H‐J , A real‐time PCR based survey on acute bee paralysis virus in German bee colonies. Apidologie 39:650–661 (2008).

[15] Cox‐Foster DL , Conlan S , Holmes EC , Palacios G , Evans JD , Moran NA *et al*, A metagenomic survey of microbes in honey bee colony collapse disorder. Science 318:283–287 (2007). 17823314 10.1126/science.1146498

[16] Blanchard P , Ribiere M , Celle O , Lallemand P , Schurr F , Olivier V *et al*, Evaluation of a real‐time two‐step RT‐PCR assay for quantitation of chronic bee paralysis virus (CBPV) genome in experimentally infected bee tissues and in life stages of a symptomatic colony. J Virol Meth 141:7–13 (2007). 10.1016/j.jviromet.2006.11.021 17166598

[17] de Miranda J and Fries I , Venereal and vertical transmission of deformed wing virus in honeybees (*Apis mellifera* L.). J Invertebr Pathol 98:184–189 (2008). 18358488 10.1016/j.jip.2008.02.004

[18] Evans J , Aronstein K , Chen Y , Hetru C , Imler JL , Jiang H *et al*, Immune pathways and defence mechanisms in honey bees *Apis mellifera* . Insect Mol Biol 15:645–656 (2006). 17069638 10.1111/j.1365-2583.2006.00682.x PMC1847501

[19] Schoning R and Schmuck R , Analytical determination of imidacloprid and relevant metabolite residues by LC MS/MS. Bull Insectol 56:41–50 (2003).

[20] Imdorf A , Buehlmann G , Gerig L , Kilchenmann V and Wille H , A test of the method of estimation of brood areas and number of worker bees in free‐flying colonies (Liebefeld method). Apidologie 18:137–146 (1987).

[21] Rasch B , Friese M , Hofmann WJ and Naumann E , Quantitative Methoden 1. Einführung in die Statistik für Psychologen und Sozialwissenschaftler. Springer‐Verlag, Berlin/Heidelberg, Germany (2009).

[22] Pituch KA , Whittaker TA and Stevens JP , Intermediate Statistics: A Modern Approach. Routledge, New York, NY (2015).

[23] Stevens J , Intermediate Statistics: A Modern Approach. Lawrence Erlbaum Associates, Mahwah, NJ (1999).

[24] Faul F , Erdfelder E , Lang A‐G and Buchner A , G* Power 3: A flexible statistical power analysis program for the social, behavioral, and biomedical sciences. Behav Res Meth 39:175–191 (2007). 10.3758/bf03193146 17695343

[25] Rasch B , Quantitative Methoden 2. Einführung in die Statistik für Psychologen und Sozialwissenschaftler. Springer‐Verlag, Berlin/Heidelberg, Germany (2014).

[26] Delucchi KL and Bostrom A , Methods for analysis of skewed data distributions in psychiatric clinical studies: working with many zero values. Am J Psychiatry 161:1159–1168 (2004). 15229044 10.1176/appi.ajp.161.7.1159

[27] Livak KJ and Schmittgen TD , Analysis of relative gene expression data using real‐time quantitative PCR and the $2^{-\Delta \Delta C_{t}}$ method. Methods 25:402–408 (2001). 11846609 10.1006/meth.2001.1262

[28] Škerl MIS , Bolta ŠV , Česnik HB and Gregorc A , Residues of pesticides in honeybee (*Apis mellifera carnica*) bee bread and in pollen loads from treated apple orchards. Bull Environ Contam Toxicol 83:374–377 (2009). 19434347 10.1007/s00128-009-9762-0

[29] Rosenkranz P , von der Ohe W , Moritz R , Genersch E , Büchler R , Berg S *et al*, Zwischenbericht eingereicht bei der Bundesanstalt für Landwirtschaft und Ernährung (BLE) Deutsches Bienenmonitoring – ‘DeBiMo’ Projektzeitraum: 01/2014–12/2014. [Online]. Available: https://bienenmonitoring.uni‐hohenheim.de/fileadmin/einrichtungen/bienenmonitoring/Dokumente/Zwischenbericht_DeBiMo_1‐12_2014.pdf [27 October 2016].

[30] Pohorecka K , Skubida P , Miszczak A , Semkiw P , Sikorski P , Zagibajło K *et al*, Residues of neonicotinoid insecticides in bee collected plant materials from oilseed rape crops and their effect on bee colonies. J Apic Sci 56:115–134 (2012).

[31] Mullin CA , Frazier M , Frazier JL , Ashcraft S , Simonds R and Pettis JS , High levels of miticides and agrochemicals in North American apiaries: implications for honey bee health. PLoS ONE 5:e9754 (2010). 10.1371/journal.pone.0009754 PMC2841636 20333298

[32] van der Zee R , Gray A , Pisa L and de Rijk T , An observational study of honey bee colony winter losses and their association with *Varroa destructor*, neonicotinoids and other risk factors. PLoS ONE 10:e0131611 (2015). 10.1371/journal.pone.0131611 PMC4496033 26154346

[33] EFSA Guidance Document on the risk assessment of plant protection products on bees (*Apis mellifera*, *Bombus* spp. and solitary bees). EFSA J 11:3295 (2013). 10.2903/j.efsa.2023.7989 PMC10173852 37179655

[34] Henry M , Beguin M , Requier F , Rollin O , Odoux J‐F , Aupinel P *et al*, A common pesticide decreases foraging success and survival in honey bees. Science 336:348–350 (2012). 22461498 10.1126/science.1215039

[35] Schneider CW , Tautz J , Grünewald B and Fuchs S , RFID tracking of sublethal effects of two neonicotinoid insecticides on the foraging behavior of *Apis mellifera* . PLoS ONE 7:e30023 (2012). 10.1371/journal.pone.0030023 PMC3256199 22253863

[36] Yang E , Chuang Y , Chen Y and Chang L , Abnormal foraging behavior induced by sublethal dosage of imidacloprid in the honey bee (*Hymenoptera: Apidae*). J Econ Entomol 101:1743–1748 (2008). 19133451 10.1603/0022-0493-101.6.1743

[37] Van der Sluijs JP , Simon‐Delso N , Goulson D , Maxim L , Bonmatin J‐M and Belzunces LP , Neonicotinoids, bee disorders and the sustainability of pollinator services. Curr Opin Environ Sustain 5:293–305 (2013).

[38] Nakagawa S and Cuthill IC , Effect size, confidence interval and statistical significance: a practical guide for biologists. Biol Rev 82:591–605 (2007). 17944619 10.1111/j.1469-185X.2007.00027.x

[39] Sandrock C , Tanadini M , Tanadini LG , Fauser‐Misslin A , Potts SG and Neumann P , Impact of chronic neonicotinoid exposure on honeybee colony performance and queen supersedure. PLoS ONE 9:e103592 (2014). 10.1371/journal.pone.0103592 PMC4118897 25084279

[40] Williams GR , Troxler A , Retschnig G , Roth K , Yanez O , Shutler D *et al*, Neonicotinoid pesticides severely affect honey bee queens. Sci Rep 5:14 621 (2015). 10.1038/srep14621 PMC4602226 26459072

[41] Whitehorn PR , O'Connor S , Wackers FL and Goulson D , Neonicotinoid pesticide reduces bumble bee colony growth and queen production. Science 336:351–352 (2012). 22461500 10.1126/science.1215025

[42] Scholer J and Krischik V , Chronic exposure of imidacloprid and clothianidin reduce queen survival, foraging, and nectar storing in colonies of *Bombus impatiens* . PLoS ONE 9:e91573 (2014). 10.1371/journal.pone.0091573 PMC3958374 24643057

[43] Goulson D , Neonicotinoids impact bumblebee colony fitness in the field; a reanalysis of the UK's Food & Environment Research Agency 2012 experiment. PeerJ 3:e854 (2015). 10.7717/peerj.854 PMC4375969 25825679

[44] Fauser‐Misslin A , Sadd BM , Neumann P and Sandrock C , Influence of combined pesticide and parasite exposure on bumblebee colony traits in the laboratory. J Appl Ecol 51:450–459 (2014).

[45] Henry M , Cerrutti N , Aupinel P , Decourtye A , Gayrard M , Odoux J‐F *et al*, Reconciling laboratory and field assessments of neonicotinoid toxicity to honeybees. Proc R Soc B 282:20152110 (2015). 10.1098/rspb.2015.2110 PMC4685821 26582026

[46] du Rand EE , Smit S , Beukes M , Apostolides Z , Pirk CW and Nicolson SW , Detoxification mechanisms of honey bees (*Apis mellifera*) resulting in tolerance of dietary nicotine. Sci Rep 5:11779 (2015). 26134631 10.1038/srep11779 PMC4488760

[47] Johnson RM , Honey bee toxicology. Annu Rev Entomol 60:415–434 (2015). 25341092 10.1146/annurev-ento-011613-162005

[48] Berenbaum MR and Johnson RM , Xenobiotic detoxification pathways in honey bees. Curr Opin Insect Sci 10:51–58 (2015). 10.1016/j.cois.2015.03.005 29588014

[49] Sanchez‐Bayo F and Goka K , Pesticide residues and bees – a risk assessment. PLoS ONE 9:e94482 (2014). 10.1371/journal.pone.0094482 PMC3981812 24718419

[50] Fischer D and Moriarty T , Pesticide Risk Assessment for Pollinators. John Wiley & Sons, Inc., New York, NY (2014).

[51] Guideline for the efficacy evaluation of plant protection products – side effects on honeybees. Bull OEPP/EPPO Bull 40:313–319 (2010).

[52] Scientific opinion on the science behind the development of a risk assessment of plant protection products on bees (*Apis mellifera*, *Bombus* spp. and solitary bees). EFSA J 10:275 (2012). 10.2903/j.efsa.2023.7989 PMC10173852 37179655

[53] Woodcock BA , Heard MS , Jitlal MS , Rundlöf M , Bullock JM , Shore RF *et al*, Replication, effect sizes and identifying the biological impacts of pesticides on bees under field conditions. J Appl Ecol 53:1358–1362 (2016).

[54] Oomen P , de Ruijter A and Steen J , Method for honeybee brood feeding tests with insect growth‐regulating insecticides. EPPO Bull 22:613–616 (1992).

[55] Pettis JS , Johnson J and Dively G , Pesticide exposure in honey bees results in increased levels of the gut pathogen *Nosema* . Naturwissenschaften 99:153–158 (2012). 22246149 10.1007/s00114-011-0881-1 PMC3264871

[56] Cresswell JE , A meta‐analysis of experiments testing the effects of a neonicotinoid insecticide (imidacloprid) on honey bees. Ecotoxicology 20:149–157 (2011). 21080222 10.1007/s10646-010-0566-0

[57] Galloway T and Handy R , Immunotoxicity of organophosphorous pesticides. Ecotoxicology 12:345–363 (2003). 12739880 10.1023/a:1022579416322

[58] Desneux N , Decourtye A and Delpuech J‐M , The sublethal effects of pesticides on beneficial arthropods. Annu Rev Entomol 52:81–106 (2007). 16842032 10.1146/annurev.ento.52.110405.091440

[59] Galloway TS and Depledge MH , Immunotoxicity in invertebrates: measurement and ecotoxicological relevance. Ecotoxicology 10:5–23 (2001). 11227817 10.1023/a:1008939520263

[60] Vanengelsdorp D and Meixner MD , A historical review of managed honey bee populations in Europe and the United States and the factors that may affect them. J Invertebr Pathol 103(Suppl. 1):S80–S95 (2010). 19909973 10.1016/j.jip.2009.06.011

[61] Di Prisco G , Cavaliere V , Annoscia D , Varricchio P , Caprio E , Nazzi F *et al*, Neonicotinoid clothianidin adversely affects insect immunity and promotes replication of a viral pathogen in honey bees. Proc Natl Acad Sci USA 110:18 466–18 471 (2013). 10.1073/pnas.1314923110 PMC3831983 24145453

[62] Alaux C , Brunet JL , Dussaubat C , Mondet F , Tchamitchan S , Cousin M *et al*, Interactions between *Nosema* microspores and a neonicotinoid weaken honeybees (*Apis mellifera*). Environ Microbiol 12:774–782 (2010). 20050872 10.1111/j.1462-2920.2009.02123.x PMC2847190

[63] Wu JY , Smart MD , Anelli CM and Sheppard WS , Honey bees (*Apis mellifera*) reared in brood combs containing high levels of pesticide residues exhibit increased susceptibility to *Nosema* (*Microsporidia*) infection. J Invertebr Pathol 109:326–329 (2012). 22285445 10.1016/j.jip.2012.01.005

[64] Retschnig G , Williams GR , Odemer R , Boltin J , Di Poto C , Mehmann MM *et al*, Effects, but no interactions, of ubiquitous pesticide and parasite stressors on honey bee (*Apis mellifera*) lifespan and behaviour in a colony environment. Environ Microbiol 17:4322–4331 (2015). 25728008 10.1111/1462-2920.12825

[65] Brandt A , Gorenflo A , Siede R , Meixner M and Büchler R , The neonicotinoids thiacloprid, imidacloprid, and clothianidin affect the immunocompetence of honey bees (*Apis mellifera* L.). J Insect Physiol 86:40–47 (2016). 26776096 10.1016/j.jinsphys.2016.01.001

[66] Lavine M and Strand M , Insect hemocytes and their role in immunity. Insect Biochem Mol 32:1295–1309 (2002). 10.1016/s0965-1748(02)00092-9 12225920

[67] Casteels P , Ampe C , Jacobs F and Tempst P , Functional and chemical characterization of Hymenoptaecin, an antibacterial polypeptide that is infection‐inducible in the honeybee (*Apis mellifera*). Biol Chem 268:7044–7054 (1993). 8463238

[68] Siede R , Meixner MD and Büchler R , Comparison of transcriptional changes of immune genes to experimental challenge in the honey bee (*Apis mellifera*). J Apic Res 51:320–328 (2012).

